# JAK Inhibitors and B Cell Function: A Comparative Study of Their Impact on Plasma Cell Differentiation, Cytokine Production, and Naïve B Cell Activation

**DOI:** 10.1002/eji.202451437

**Published:** 2025-03-17

**Authors:** Wenqi Huang, Charlotte de Vries, Ravi Kumar Sharma, Kittikorn Wangriatisak, Katerina Chatzidionysiou, Vivianne Malmström, Caroline Grönwall

**Affiliations:** ^1^ Division of Rheumatology, Department of Medicine Solna, Karolinska Institutet, Center for Molecular Medicine Karolinska University Hospital Stockholm Sweden; ^2^ Department of Clinical Immunology and Rheumatology All India Institute of Medical Sciences Bilaspur India

**Keywords:** B‐lymphocytes, JAK inhibitor, tofacitinib, baricitinib, upadacitinib, filgotinib

## Abstract

B cells play a crucial role in autoimmune diseases, as evidenced by autoantibody responses and the effectiveness of B cell‐targeted therapies. Janus kinase inhibitors (JAKi), which target downstream signaling of cytokine receptors, are potent rheumatic disease‐modifying drugs. However, besides reducing inflammation, JAKi may impact the adaptive immune system. In this study, we examined the effects of JAKi on B‐cell function using in vitro cultures and multiparameter flow cytometry. The results show a JAKi‐mediated reduction in plasma cell differentiation, primarily by inhibition of memory B‐cell stimulation and proliferation. JAKi exposure resulted in stalling R848, IL‐2, and IL‐21 stimulated B cells in an intermediate activated state with elevated naïve cells displaying increased expression of CXCR5, CD71, CD22, and CD20. In addition, the data demonstrate a moderate JAKi‐mediated reduction of B cell TNF and IL‐8 cytokine expression following stimulation. Importantly, the efficacy varied greatly between drugs; tofacitinib and upadacitinib (pan JAKi; JAK1i) exhibited the strongest impact, while baricitinib (JAK1/JAK2i) showed donor‐dependent variation, and filgotinib (JAK1i) had no effect. All JAKi, except filgotinib, inhibited IL‐2 or IL‐21‐induced STAT3 phosphorylation. Still, filgotinib demonstrated similar inhibition of phospho‐STAT5 as other JAKi following IL‐21. These findings underscore the therapeutic impact of JAKi through the modulation of B‐cell functions.

## Graphical abstract

JAK inhibitors (baricitinib, tofacitinib, upadacitinib), except for filgotinib, significantly reduce plasmablast differentiation but only partly inhibit activation of naïve B cells and exert a more modest effect on TNF and IL‐8 production. Unlike other JAK inhibitors, filgotinib selectively inhibits STAT5 phosphorylation without affecting STAT3 phosphorylation in response to IL‐21.

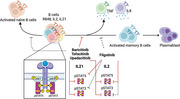

## 1 Introduction

B cells are key players in numerous autoimmune and chronic inflammatory diseases associated with autoantibodies and MHC class II genetic predisposition, which is also underscored by the efficacy of B cell targeted therapy [1], Notably, B cells can influence autoimmune diseases through the production of autoantibodies and cytokines as well as via antigen presentation and subsequent T cell activation. Moreover, elevation of cytokines contributes substantially to the pathogenesis of diseases such as rheumatoid arthritis (RA) and systemic lupus erythematosus (SLE). Hence, strategies for blocking cytokines or cytokine receptors are commonly used therapies, for example, blockade of tumor necrosis factor (TNF), interleukin 6 receptor (IL‐6R), or type one interferon receptor (IFNR). While these drugs can have high efficacy, they are postulated to primarily inhibit the effector phase of the disease, presumably with a more limited effect on the adaptive immune system and the underlying autoimmunity. A recent group of targeted synthetic disease‐modifying anti‐rheumatic drugs (tsDMARDs) comprises the Janus kinase inhibitors (JAKis), which have been shown to be efficacious in RA [2].

The JAK family is composed of nonreceptor tyrosine protein kinases, which are intracellularly associated with various cytokine and growth hormone receptors and mediate binding and phosphorylation of signal transducer and activator of transcription (STAT) proteins, leading to STAT targeted regulation of gene transcription. The JAK family consists of four main members: JAK1, JAK2, JAK3, and TYK2, which regulate the signaling of different cytokine receptors and interact with different STATs. Dysregulation of cytokines and the JAK/STAT signaling pathway is associated with several diseases, and different oral JAKis have been approved for the treatment of RA, psoriatic arthritis, ankylosing spondylitis, atopic dermatitis, and ulcerative colitis [3]. The JAKis baricitinib, filgotinib, tofacitinib, and upadacitinib are used in clinical practice for the treatment of RA in Europe [3]. JAK inhibition represents an effective treatment option, yet there have also been safety signals indicating a small but significant risk of thrombosis and cardiovascular disease [4, 5]. While the available drugs belong to the same category, they have different JAK target selectivity and may exhibit different efficacy and safety profiles [5, 6, 7, 8]. Tofacitinib was the first JAKi drug to be developed and primarily inhibits JAK1/JAK3 with lesser effect on JAK2, while the second drug on the market, baricitinib, is a JAK1/JAK2 inhibitor, and the subsequent upadacitinib and filgotinib are considered second generation inhibitors due to higher selectivity for JAK1 [9, 10, 11, 12]. Compared with other JAKis, JAK1 inhibitors show fewer hematological effects and less unwanted immune suppression, as evidenced by reduced virus reactivation in the context of herpes zoster [7].

As cytokines orchestrate B cell differentiation and activation, JAK inhibition is likely not only limiting cytokine signaling but also affecting B cell functions. In this study, we have used different in vitro B cell assays for evaluating the effect of a variety of JAK inhibitors. Analysis was conducted using multiparameter spectral flow cytometry to examine changes in B‐cell surface markers and intracellular cytokine expression.

## 2 Results

### 2.1 JAK Inhibitors Limit Plasmablast Differentiation

**FIGURE 1:**
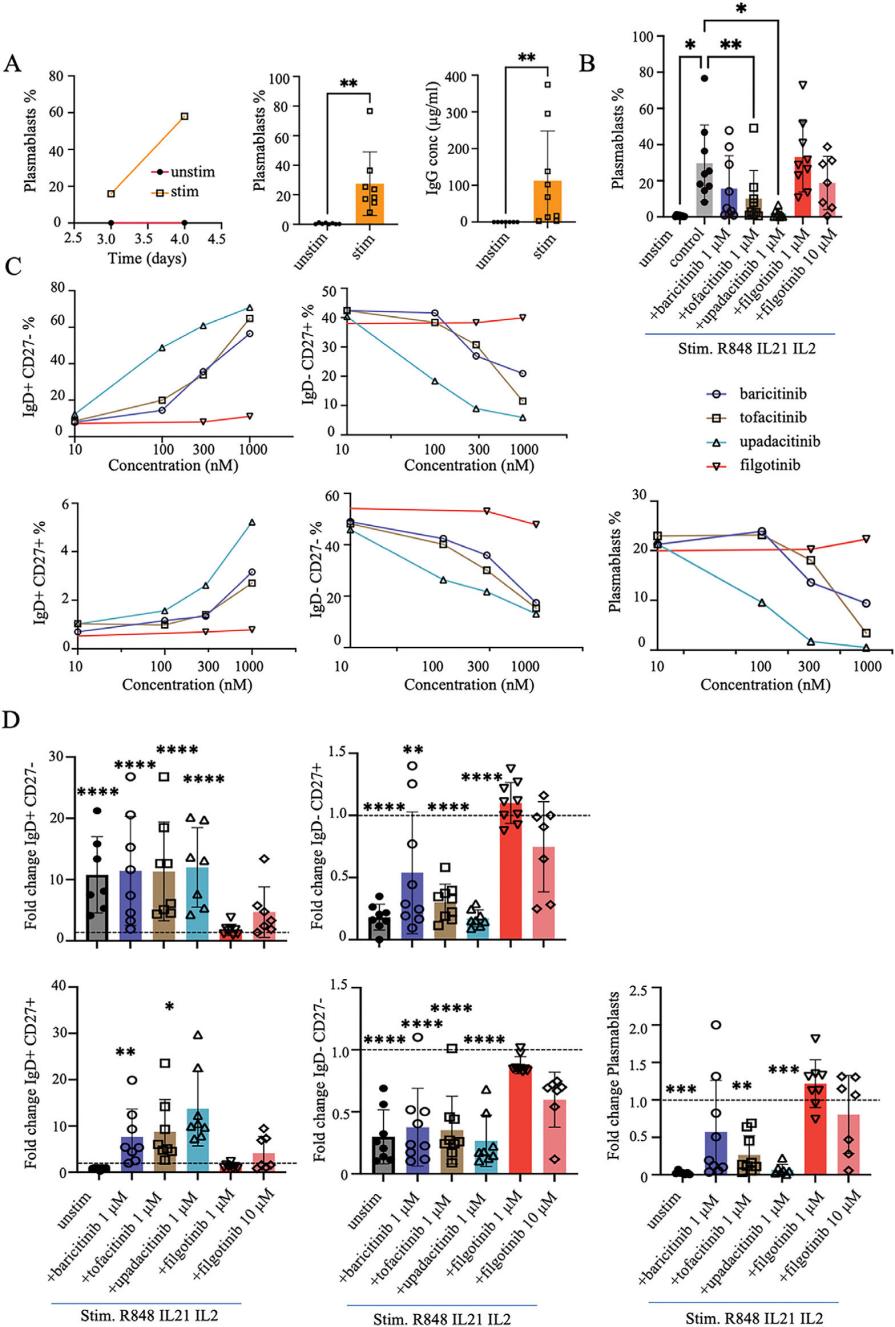
JAK inhibitors can block B cell differentiation. PBMCs from healthy donors were cultured with R848, IL‐2, and IL‐21 with and without the addition of JAKi at the indicated concentration. (A) Percentage of plasmablast by flow cytometry among CD19+ B cells after 3‐ or 4‐day stimulation (left panel). Percentage of plasmablast by flow cytometry (middle) and IgG expression in the supernatants by ELISA (right) comparing four days of culturing with and without stimulation (*n* = 9). (B) Overall proportion of plasmablasts among CD19^+^ B cells in unstimulated cells, control stimulated cells, and cells stimulated in the presence of different JAK inhibitors. (C) Proportion of different B‐cell populations (IgD^+^ CD27^−^ naïve B cells, IgD^−^ CD27^−^ double negative B cells, IgD^−^ CD27^+^ memory B cells, IgD^+^ CD27^+^ unswitched memory B cells and CD38^high^ CD27^high^ plasmablasts) among all CD19^+^ B cells after stimulation and JAKi treatment (*n* = 8). Individual data is available in Figure S3. (D) Fold change difference of B‐cell populations in unstimulated cells or R848, IL‐2, and IL‐21 stimulated cells with the addition of different JAKis. The fold change was calculated by comparing each treatment group to the baseline control stimulated condition (R848, IL‐2, IL‐21; dashed line) (*n* = 8). Bar graphs represent the average ± standard deviation (SD). Data were compiled for four experiments with two donors per experiment. Group comparisons were analyzed by one‐way ANOVA with Tukey's multiple comparisons test. Only statistically significant *p*‐values <0.05 are presented. *p<0.05, **p<0.01 ***p<0.001, ****p<0.0001.

To investigate the effect of JAK inhibition on B cell differentiation into plasmablasts, we set up an in vitro protocol evaluating TLR7/8 agonist R848, together with IL‐2 and IL‐21 for stimulation of healthy donor PBMC (Figure 1A) based on previously reported methods [13, 14, 15]. Hereby, a robust plasmablasts population (CD27^high^ CD38^high^ cells) was generated as assessed by flow cytometry (gating strategy is showed in Figure S1). Plasmablasts were only observed after stimulation and made up 10–20% of the culture after three days and 20–60% after 4 days (*n* = 8, *p* = 0.01). This was also confirmed by total IgG measurements in the supernatant, which was increased by day 4 (Figure 1A).

We selected four JAK inhibitors for our study: baricitinib, tofacitinib, upadacitinib, and filgotinib, which were used at concentrations ranging from 10 to 1000 nM, that is, within an equivalent range of therapeutic dosing and without a significant effect on cell viability [16, 17, 18] (Figure S2). Compared with stimulation only (with added DMSO), we could observe a significant dose‐dependent inhibitory effect of upadacitinib, tofacitinib, and baricitinib on plasmablast differentiation (Figure 1B,C; Figure S3). Upadacitinib at 1000 nM had the strongest inhibitory effect, with a 19‐fold decrease in the frequency of plasmablasts compared with the control‐stimulated cells. Tofacitinib and baricitinib at 1000 nM had less effect, with a fourfold and twofold decrease in plasmablasts, respectively (Figure 1C,D). The effect of tofacitinib and baricitinib showed larger interdonor variability, which may contribute to the lower average efficacy (Figure 1B–D). Dose‐dependent effects were observed, shown as average values in Figure 1 and as individual values in Figure S3. The effect was significant both when assessing the absolute frequencies presented in Figure S3 and the fold change differences in Figure 1C. Interestingly, filgotinib at 1000 nM had no effect. A weak trend for decrease could be seen when using filgotinib at 10 µM, but without reaching statistical significance (Figure 1D).

Assessment of IgG and IgA production showed that, as expected, antibody levels decreased in the presence of tofacitinib, baricitinib, and upadacitinib in a dose‐dependent manner. Filgotinib exhibited no significant impact on IgG and IgA concentrations (Figure S4D,E). Moreover, adding the JAK inhibitors at day 3 of the cultures did not significantly affect the immunoglobulin productions, emphasizing the primary effect on B cell differentiation (Figure S4F).

### 2.2 JAK Inhibitors Mediate a Shift of B Cell Populations in Stimulated Cultures

In addition to the elevation of plasmablasts in the cultures, the in vitro stimulation condition with R848, IL‐2, and IL‐21 caused general alterations in the phenotypic composition of B cells due to both differentiation and proliferation of different B cell subsets (Figure S4). Higher frequencies of both CD27^+^ IgD^−^ memory B cells and double negative CD27^−^ IgD^−^ (DN) B cells were observed after stimulation (Figure S4). With the addition of baricitinib, tofacitinib, and upadacitinib, these population shifts were attenuated, hence, a lower frequency of CD19^+^ IgD^−^ CD27^+^ memory B cells and CD27^−^ IgD^−^ DN B cells were observed compared with the control stimulated group without JAKi (Figure 1; Figure S3B,D). Correspondingly, the frequencies of IgD^+^ CD27^+^ B cells and naïve IgD^+^ CD27^−^ B cells were higher in the stimulated cultures with baricitinib, tofacitinib, and upadacitinib (Figure 1, Figure S3C; Figure S4A). In addition, JAKi treatment resulted in an approximately 1.4‐fold decrease in the overall frequency of B cells, except for filgotinib, which did not exhibit any effect. Upadacitinib, which showed the strongest effect in inhibiting plasmablasts differentiation, also had a strong effect in decreasing memory B cells and DN B cells (6.5 and 4‐fold, respectively). Similarly, tofacitinib gave a three‐ and fourfold decrease in the frequency of memory B cells and DN B cells, respectively. Baricitinib had a weaker effect on the frequency of memory B cells and DN B cells (two and threefold decrease, respectively), and filgotinib did not show any effect (Figure 1D).

### 2.3 Filgotinib Demonstrates Inhibition of STAT5 but Not STAT3 Phosphorylation Following IL‐21

**FIGURE 2:**
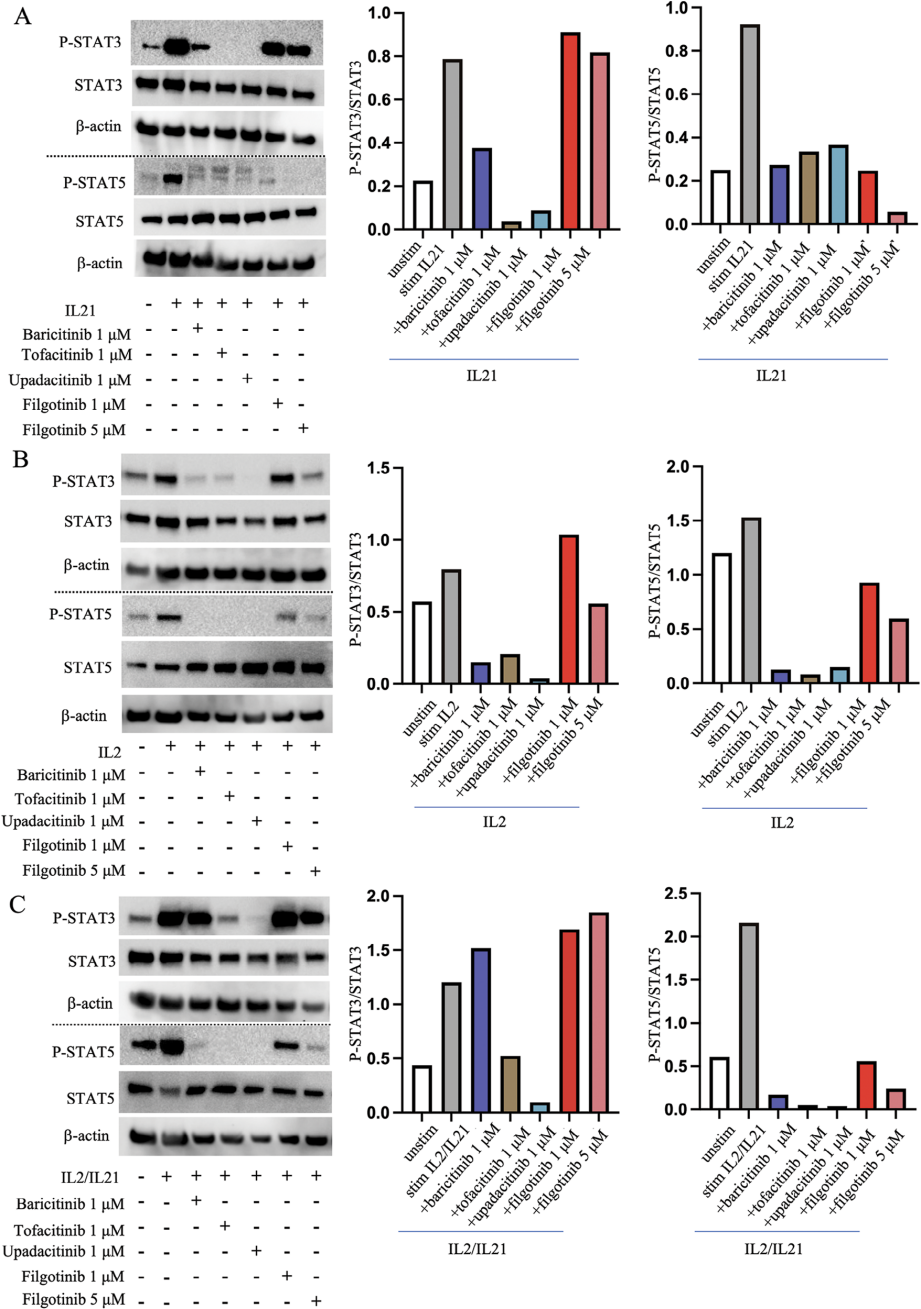
JAK inhibitors display differences in ability to inhibit STAT3 versus STAT5 phosphorylation following IL‐2/IL‐21. Western blot analysis of pSTAT3/ STAT3/ pSTAT5/ STAT5 from purified B cells after 20 min IL‐2 (A), IL‐21 (B), or IL‐2+IL‐21 (C) stimulation. Purified B cells, 1 × 10^6^ per condition, were isolated from PBMCs and given 1 µg/ml R848 incubation with/without different JAKi treatment overnight before cytokine stimulation. Cell lysate was analyzed by Western blot, and changes in pSTAT3, STAT3, pSTAT5, and STAT5 were assessed. β‐Actin was used for additional loading control in all experiments.

Next, we sought to investigate the effects of the JAK inhibitors on downstream STAT phosphorylation. Purified peripheral B cells were stimulated with IL‐21 and/or IL‐2, which resulted in STAT3 and STAT5 phosphorylation to different extents as assessed by Western blot (Figure S5). All JAK inhibitors, including filgotinib, suppressed B‐cell IL‐21 or IL‐2‐induced STAT5 phosphorylation, although higher concentrations of filgotinib were required to suppress IL‐2‐induced pSTAT5 (Figure 2A–C; Figure S6). IL‐2‐induced pSTAT3 phosphorylation was inhibited by baricitinib, tofacitinib, and upadacitinib, and while a trend was seen for filgotinib at 5 µM, it could not be validated when normalizing for total STAT3 expression (Figure 2B). Moreover, filgotinib at both 1 and 5 µM concentrations failed to inhibit IL‐21‐induced STAT3 phosphorylation, and baricitinib demonstrated a weaker inhibitory effect compared with tofacitinib and upadacitinib (Figure 2A). Under combined IL‐2 and IL‐21 stimulation, the inhibitory trends resembled those of IL‐21‐induced signaling, with baricitinib not showing any inhibitory effect on STAT3 but significant inhibition of STAT5 phosphorylation (Figure 2A–C). Filgotinib did not display any significant inhibition of STAT3 but moderate inhibition of STAT5 phosphorylation in the IL‐2/IL‐21 combined stimulation. These results, in particular the selective effects of filgotinib, may implicate that of the assessed pathways, the IL‐21‐induced STAT3 signaling is most critical for B cell differentiation.

### 2.4 Effect of JAK Inhibitors on Naïve B Cell Activation

**FIGURE 3:**
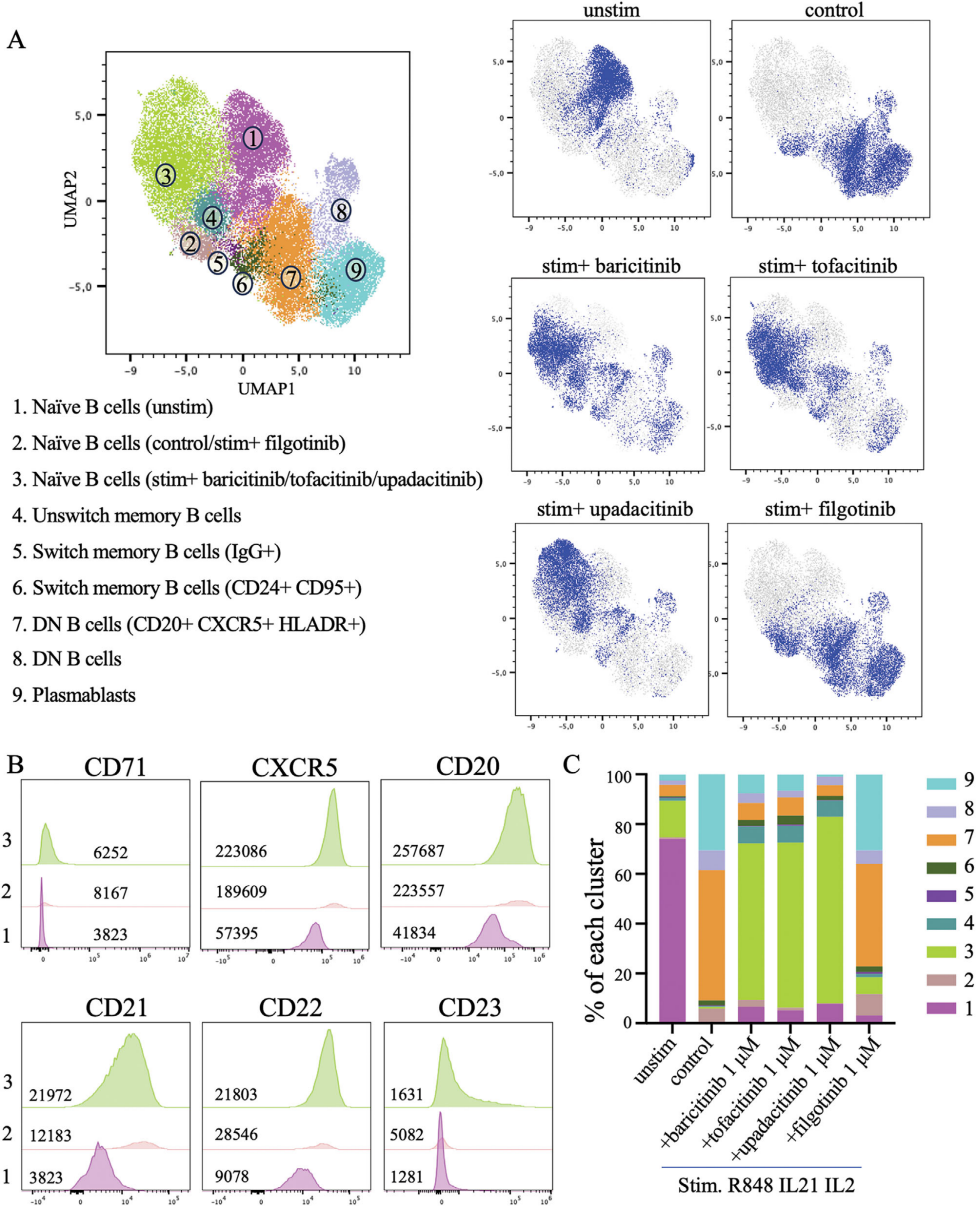
JAK inhibition results in shifts of B cell populations after stimulation. PBMCs from healthy donors were cultured with R848, IL‐2, and IL‐21 with and without the addition of JAKi, and the B cell phenotype was assessed by spectral flow cytometry analysis. (A) FlowSOM analysis and UMAP visualization of different stimulation conditions, comparing unstimulated cells with cells stimulated with R848, IL‐21, and IL‐2 in the presence of JAKi upadacitinib 1 µM, baricitnib 1 µM, tofacitnib 1 µM, and filgotinib 1 µM. The stimulated control condition had R848, IL‐21, and IL‐2 with DMSO. A total of 2000 cells were selected from all groups. (*n* = 4) (B) Different marker (CD71, CXCR5, CD20, CD21, CD22, CD23) expression in clusters 1, 2, and 3. The numbers shown in the histogram are median valued for the mean fluorescent intensity (MFI) of the markers in each cluster for the donors. (C) Percentage of B cell clusters after different culturing conditions (*n* = 4).

Flow cytometry data from live CD3^−^/CD14^−^/CD16^−^/CD19^+^ cell populations were dimensionally reduced by Uniform Manifold Approximation and Projection (UMAP) and clustered by FlowSOM (Figure 3A, *n* = 4). The majority of the unstimulated B cells were naïve (Figure 3C, cluster 1), and following stimulation, most naïve B cells reside in cluster 2. However, in the presence of baricitinib, tofacitinib, and upadacitinib, these cells shifted to cluster 3. Cluster 3 represented an activated phenotype with significantly increased expression of the proliferation marker CD71 and the activation marker CXCR5, as well as general B cell markers such as CD20, CD21, CD22, and CD23, compared with cluster 1 (Figure 3B; Figure S7). Hence, we interpret that the accumulation of naïve B cells with an activated phenotype, but no further differentiation to memory or plasma cells, implicates that the B cells get stalled in an intermediate activated state in the presence of JAKi. In contrast, exposure to filgotinib did not result in a phenotype change in the naïve B cells compared with the stimulation control, which was reflected by a similar predominance of cluster 2.

**FIGURE 4:**
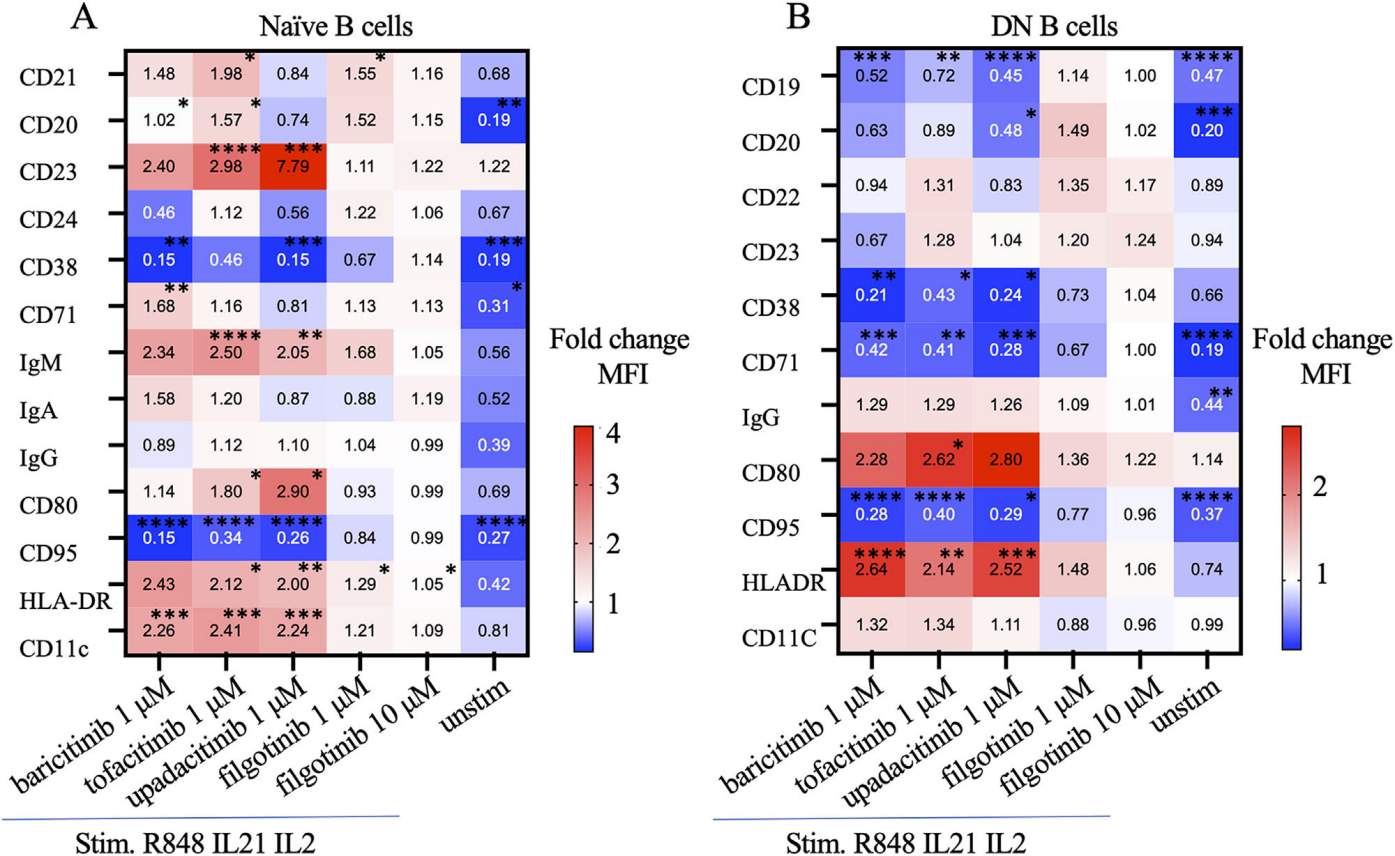
JAK inhibitors mediate change in surface markers in naïve and double negative B cells after stimulation. Assessment of shifts in surface markers in stimulated cells with and without the addition of JAKi by fold change comparison of cell surface markers (MFI value) in naïve B cells or double negative cells. Conditions of R848, IL‐21, and IL‐2 stimulation with different JAKi were compared with the stimulated control group (R848, IL‐21, IL‐2, and DMSO) in naïve B cells (left) or in double negative B cells (right). The numbers indicate fold change difference in MFI of markers of naïve B cells or DN B cells in JAKi treatment groups and unstimulated group compared with stimulated groups. Red color represents increase (fold change >1) while blue represents decrease (fold change <1) compared with the with control stimulated group. MFI value (mean ± SD) in different cell subset comparisons were by one‐way ANOVA with Tukey's multiple comparisons test. Only statistically significant *p*‐values <0.05 are presented. *p < 0.05, **p < 0.01 ***p < 0.001 ****p < 0.0001.

Using conventional manual gating, we could detect differences in surface markers on CD27‐ IgD+ naïve B cells and CD27^−^ IgD^−^ double negative B cells in JAKi treated stimulated cells (baricitinib, tofacitinib and upadacitinib) compared with control stimulated cells, with the most consistent being an increase in CD11c, CD23, IgM and HLA‐DR mean fluorescence intensity (MFI) expression following baricitinib, tofacitinib and upadacitinib (Figure 4A,B). In contrast, CD95 expression, which was upregulated after stimulation, was lower in the JAKi treatment groups compared with the control stimulated group in naïve, DN, and SM B cells, while filgotinib did not have the same effect (Figure 4A,B; Figure S8). Within DN and SM B cells, the markers CD71 and CD38 had similar trends as CD95 between groups (Figure 4A,B; Figure S8).

We also investigated the influence of JAKi on naïve B cells after BCR‐activation to understand the possible impact of the antigen‐presenting ability of the B cells. Hence, we stimulated the cells with anti‐IgM and IL‐21 to mimic antigen‐mediated activation of naïve cells. With this stimulation, we observed an increase in the expression of the surface markers CXCR5, CD95, CD11c, and CD21 on naïve B cells, along with a slight increase in HLA‐DR expression (Figure S9C). The addition of upadacitinib, baricitinib, or tofacitinib at 1000 nM led to a downward trend in the MFI of CD95, with statistically significant downregulation observed for CXCR5 CD11c and CD21 (Figure S9C). In contrast, HLA‐DR expression showed statistically significant upregulation following JAKi exposure. Notably, filgotinib had no impact on the analyzed markers. Anti‐IgM naïve B‐cell activation did not result in any significant shifts of traditional B cell subsets or result in plasma cell differentiation, and hence these B cell subsets were not significantly impacted by JAKi (Figure S10). Yet, analysis using UMAP revealed differences in naïve B cell clusters between stimulated and unstimulated states, with JAK inhibitors, except for filgotinib, resulting in clusters distinct from stimulated cell states (Figure S9A).

### 2.5 Effects of JAK Inhibitors on TNF and IL‐8 Production

While the main mechanism of JAK inhibitors is the blocking of downstream cytokine signaling, we also wanted to understand if JAK inhibitors would affect the secretion of TNF and IL‐8 by B cells. TNF and IL‐8 are proinflammatory cytokines, which autoreactive and disease‐associated B cells have been suggested to be able to express [19, 20]. We used the same culture conditions as described previously to prime the cells but with shorter time points and with the addition of PMA, ionomycin, and BFA for the last four hours of culture to induce cytokine expression, which was assessed by intracellular flow cytometry (see Figure S11 for gating strategy). Upon stimulation, the numbers of TNF^+^ and IL‐8^+^ B cells peaked at 6 h and subsequently decreased (Figure S11B). Notably, PMA, ionomycin, and BFA alone without R848, IL‐21, and IL‐2 did not result in cytokine expression.

**FIGURE 5:**
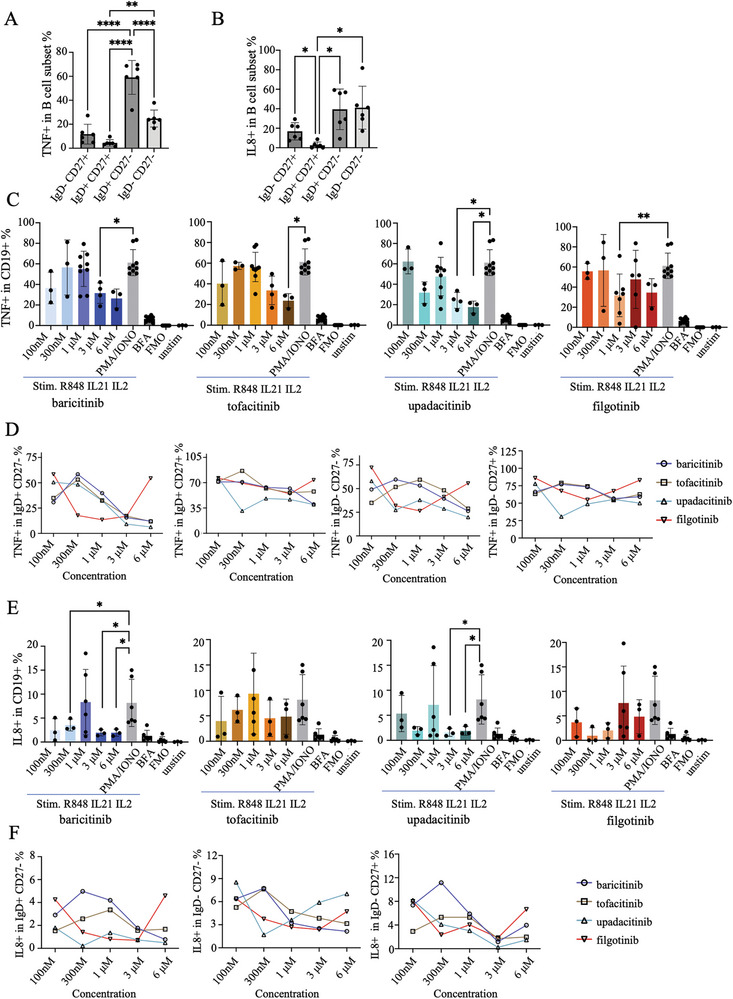
Effect of JAK inhibitors on B cell expression of IL‐8 and TNF. PBMCs were primed with R848, IL‐21, IL‐2, and subsequent of phorbol 12‐myristate 13‐acetate (PMA), ionomycin (IONO), and Brefeldin A (BFA) to induce intracellular expression of cytokines. (A, B) Percentage of TNF+ and IL‐8+ B cells in different B cell subsets. (C) After 6 h stimulation, the percentage of IgD^+^ CD27^−^ TNF^+^ B cells; IgD^+^ CD27^+^ TNF^+^ B cells; IgD^−^ CD27^−^ TNF + B cells; IgD^−^ CD27^+^ TNF^+^ B cells after the addition of different JAK inhibitors at indicated concentrations. (D) After 6 h stimulation, percentage of TNF^+^ B cell after addition of different JAK inhibitors at indicated concentrations. (E) After 6 h stimulation, the percentage of IgD^+^ CD27^−^ IL‐8^+^ B cells; IgD^−^ CD27^−^ IL‐8^+^ B cells; IgD^−^ CD27^+^ IL‐8^+^ B cells after the addition of different JAK inhibitors at indicated concentrations. (F) After 6 h stimulation, percentage of IL‐8^+^ B cell percentage under different JAK inhibitors at indicated concentrations. Stim: R848, IL‐2, IL‐21 stimulation; PMA/IONO: PMA, ionomycin, brefeldin A; FMO, Fluorescence Minus One: Control without the TNF or IL‐8 staining; Stim+BFA: After R848, IL‐2, IL‐21 stimulation, only BFA was added for further stimulation. Data were compiled for two experiments with three donors per experiment. Bar graphs represent mean ± SD. Group comparisons were analyzed by one‐way ANOVA with Dunnett's correction for multiple comparisons. Only statistically significant *p*‐values <0.05 are presented. *p < 0.05, **p < 0.01, ***p < 0.001, ****p < 0.0001.

Both baricitinib and tofacitinib were found to exhibit a significant inhibitory effect on TNF production at 3000 and 6000 nM but no effect at concentrations below 1000 nM. Upadacitinib displayed a noticeable inhibitory effect on TNF^+^ B cells at concentrations of 300, 3000, and 6000 nM, while filgotinib only exerted a weak inhibitory effect on TNF^+^ B cells (Figure 5C,D).

The prevalence of IL‐8‐producing cells among all CD19^+^ B cells was lower than for TNF (approximately 20% compared with 60% for TNF). Yet, similar to TNF expression, IL‐8 production was seen across different B cell subsets (Figure 5A). Baricitinib and upadacitinib at concentrations of 100, 300, 3000, and 6000 nM inhibited the frequency of IL‐8 positive cells after stimulation. Yet, the effects were moderate, and tofacitinib did not show any significant effect. Curiously, no effects were observed at 1000 nM JAKi, but this could be due to donor variation and that additional donors were included for this concentration (Figure 5E,F). Filgotinib exhibited an IL‐8 inhibitory effect for 100–1000 nM, but this was not dose‐dependent, and no inhibition was observed at the higher concentrations (Figure 5E,F).

## 3 Discussion

In this study, we dissect the impact of JAK inhibition on B cells using in vitro culturing and multiparameter flow cytometry. By in‐depth phenotyping, we could determine that JAKi inhibited plasma cell differentiation but did not completely inhibit activation of naïve B cells and stalled naïve B cells in an intermediate state of activation. Similarly, when using BCR stimulation and JAKi, no inhibition was seen and, if anything, JAKi resulted in higher HLA‐DR expression, suggesting no effect on B cell antigen presenting capacity. However, JAKi moderately impacted B cell expression of TNF and IL‐8 after stimulation. Importantly, our data demonstrate that JAKi have the capacity to modulate B cell function with different efficacy.

Our stimulation protocol includes a cocktail of the TLR ligand R848 with IL‐2 and IL‐21, which together induce plasmablasts differentiation and class‐switched immunoglobulin secretion, as well as prime the cells to produce cytokines. The TLR7/8 agonist represents T‐cell independent B cell activation signal and leads to downstream NF‐κB and MAPK signaling [21]. IL‐21, on the other hand, is a pleiotropic cytokine that moderates multiple B‐cell functions, including proliferation and differentiation, and IL‐2 contributes further to B‐cell differentiation [22]. Both IL‐2 and IL‐21 are derived from T cells under physiological conditions, and receptor engagement leads to JAK1/JAK3 activation [23, 24]. Hence, in this study, we expected our culture system to be dependent on JAK1. Our results showed that upadacitinib had the strongest effect, while baricitinib and tofacitinib have a moderate effect, and filgotinib had no effect on B cell activation and differentiation. This data is in line with previous reports, but our analysis provides a more in‐depth dissection of the effect of different JAKis [25, 26, 27, 28]. Compared with the data from a previous report by Frede et al. [28] using a CpG TLR9 ligand to stimulate B cells, our study showed stronger effects of JAKi on B cell differentiation, including a consistent inhibitory effect on IgG expression, with the exception of filgotinib. Furthermore, the concentration range utilized in our study was similar to that in other investigations and broadly corresponds to the physiological plasma concentrations of JAK inhibitors during treatment [27, 29]. Filgotinib has a higher IC50 and dosing regimen [30]; hence, for this JAKi, we also included a ten times higher concentration in the assays, which still did not have a significant effect. Tofacitinib and baricitinib target pathways that depend on JAK1, JAK2, and JAK3, while upadacitinib and filgotinib have selectivity for JAK1 [23, 25, 26]. Biochemical in silico analysis suggests the highest JAK1 selectivity for upadacitinib [31], but in vitro experiments have also demonstrated its ability to inhibit cytokine‐induced JAK2/3 activation [25, 26]. Differences in selectivity or IC50 may explain why JAK1i upadacitinib is different from filgotinib. Both JAK1 and JAK3 are involved in signaling through IL‐2R and IL‐21R. While IL‐21‐mediated activation of STAT5 can regulate naïve B cells [32], IL‐21‐mediated STAT3 signaling is critical for human memory B cell activation and plasma cell differentiation [23, 33]. The lack of a filgotinib inhibitory effect on STAT3 phosphorylation may explain its inability to impact the B cell differentiation. Considering the reported high selectivity of filgotinib for JAK1, the results may implicate that the JAK3‐STAT3 axis rather than JAK1‐STAT3 may be dominating downstream of the IL‐21R in B cells.

Significantly, both upadacitinib and filgotinib have good efficacy in treating RA [34, 35]. Filgotinib has also been shown to reduce cytokine expression that may affect B cells indirectly (CXCL13, IL‐7, IL‐21) in RA patients, and the treatment was associated with a slight increase in peripheral B cell numbers [36]. Regarding antibody production, patients receiving JAKi (primarily baricitinib and tofacitinib) were reported to have lower vaccine responses in one study [37], while no noticeable change was seen in another study of individuals receiving upadactinib [38]. Moreover, tofacitinib has been shown to reduce circulating levels of IgG and rheumatoid factor (RF) in RA patients but not change B cell counts [12, 39]. Yet, the activated phenotype of anti‐citrullinated protein antibody (ACPA)^+^ memory B cells in RA does not seem to change after JAKi treatment despite clinical responses [40].

In addition to plasmablast differentiation following the in vitro stimulation with R848, IL‐2, and IL‐21, we observed shifts in other B cell subsets, including an increase in DN B cells. Elevated frequencies of DN B cells are evident in individuals with various inflammatory and infectious conditions [41]. Moreover, recent studies in SLE suggest that a subset of DN B cells may originate from activated naïve B cells and subsequently differentiate into plasmablasts through extra‐follicular maturation pathways [41]. Intriguingly, compared with control‐stimulated cells, JAK inhibitors blocked DN cell formation in culture, but with a seemingly stronger effect on CXCR5^+^ DN1‐like cells (cluster 7) than on CXCR5^−^ DN cells (cluster 8), which more closely resemble disease‐associated DN2.

An important novel finding in our study is the effect of JAKi on naïve B cells. When stimulated with IL‐21, IL‐2, and R848 in the presence of JAKi, the naïve B cells still got activated as reflected by the phenotypic change compared with unstimulated naïve B cells as illustrated in unsupervised FlowSOM analysis and UMAP visualization. Activated naïve B cells are gaining increasing attention in autoimmune diseases. Prior investigations have shown that SLE patients have an increase in the frequency of activated naïve B cells, which exhibit elevated levels of CD19, CD11c, and CD23 while displaying diminished expression of CD21, CD24, and CD38 [42]. Recently, we have also found activated naïve B cells (CD21‐CXCR5‐) to be increased when comparing ACPA^+^ risk‐RA progressors to ACPA^+^ risk‐RA nonprogressors [43]. Furthermore, autoantibody‐positive established RA patients display dysregulation in naïve B cell populations [44]. In conclusion, JAKi may not significantly impact the initial activation of naïve B cells but may block further activation. It should be noted that even if JAKi may not significantly inhibit activation of naïve B cells or DN2 cells in patients, the effect on memory cells would be crucial.

We also investigated the JAKi effect on B cells cytokine expression with a focus on TNF and IL‐8. TNF is a key mediator of inflammation in RA, which is emphasized by the clinical success of TNF inhibitors [45]. The granulocyte chemoattractant IL‐8 has gained increased interest due to synovial expression and the postulated important role for neutrophils in the pathogenesis of RA [46]. Notably, both cytokines can be produced by B cells [20, 47, 48] (as well as by several other cell types), and RA‐associated autoreactive B cells have been proposed to be especially potent expressers [19]. In our assay, the effector cells were primed with R848, IL‐21, and IL‐2 stimulation followed by broad activation with PMA and ionomycin. We could observe an inhibitory effect of several JAKis. While TNF and IL‐8 expression is not directly regulated by the JAK‐STAT pathway, their expression can be influenced indirectly. However, it should be noted that the effect was moderate and only significant at higher JAKi concentrations. In conclusion, inhibition of cytokine expression was not as stable as the effect on plasmablasts’ differentiation.

Interestingly, RA has been suggested to have different subtypes based on synovial pathology, and patients may have a disease with more B cell‐ and cytokine‐driven mechanisms [49]. Hence, different JAKi, including filgotinib, may have different efficacies in different RA patient subsets depending on pathotype. Future studies are merited to further investigate which patients may benefit most from a certain JAKi drug.

In summary, our study reveals the heterogeneous impact of JAKi on B cell functionality and differentiation, which may be important in the context of treatment of autoimmune pathologies such as RA. This further points toward the need for more research for a deep understanding of the mechanism of action and on‐ and off‐target effects of different JAKi on the immune system. These observations contribute important insights into the mechanism of JAKi in autoimmune diseases, thereby enriching the understanding of their therapeutic efficacy.

## 4 Data Limitations and Perspectives

The experiments were performed in vitro with the use of a limited number of samples from healthy donors. Hence, it is uncertain how well these results can be extrapolated to RA patients, considering that RA patients have altered B‐cell responses and phenotypes. While flow cytometry allowed for specific analysis of B cells in the PBMC cultures, we acknowledge that JAK inhibitors may also affect other cell types within the PBMC population, potentially influencing B‐cell responses indirectly. Yet, the signaling experiments performed in purified B cell cultures clearly demonstrate a specific B‐cell effect.

## 5 Materials and Methods

Complete methods are provided in Supporting Information.

### 5.1 B Cell Stimulation Assays

Briefly, peripheral blood mononuclear cells (PBMC) were purified from healthy blood donors (Table S1) by Ficoll‐Paque (Cytiva) and cultured in the presence of 1 µg/mL R848 (Invivogen), 10 ng/mL human recombinant IL‐2 (Peprotech), and 50 ng/mL human recombinant IL‐21 (Peprotech) without the addition of JAKi (upadacitinib, baricitinib, tofacitinib, or filgotinib) at indicated concentrations or DMSO vehicle. Naïve B cell activation was stimulated by 3 µg/mL anti‐human‐IgM (Jackson ImmunoResearch) and 50 ng/mL IL‐21. Plasmablast differentiation and phenotypic changes were assessed after 3–4 days by spectral flow cytometry. The complete flow cytometry protocol, antibody panels (Table S2 and S3), gating strategy (Table S4, Figures S1 and S10), and analysis methodology are provided in Supporting Information. Immunoglobulin levels were assessed in supernatants by ELISA. Cytokine expression was analyzed by intracellular flow cytometry after 6 or 12 h, with the addition of phorbol 12‐myristate 13‐acetate (PMA; 50 ng/mL), ionomycin (1 µg/mL), and Brefeldin A (BFA) in the final 4 h of culture before harvest. STAT phosphorylation was assessed in purified B cells (Milteyi Biotec, B cell isolation kit II) that were rested overnight and stimulated for 20 min at 37°C before cell lysis and Western blot analysis.

## Author Contributions

Wenqi Huang, Vivianne Malmström, and Caroline Grönwall designed and planned the study and interpreted the data. WH performed experimental work. Charlotte de Vries, Ravi Kumar Sharma, and Kittikorn Wangriatisak contributed to flow cytometry, cell culturing methodology, data analysis strategies, and interpretation. Katerina Chatzidionysiou provided clinical insights and discussion. Wenqi Huang wrote the first draft of the manuscript. All authors were involved in writing the article and revising it critically. All authors participated in discussions finalizing the manuscript and approved the final version.

## Conflicts of Interest

The authors declare no conflicts of interest.

### Peer Review

The peer review history for this article is available at https://publons.com/publon/10.1002/eji.202451437

## Ethics approval statement for human and/or animal studies

All subjects gave informed consent, and the study was approved by local ethics review committees.

## Supporting information



Supporting information

## Data Availability

The data supporting the findings of this study are available from the corresponding author upon reasonable request.
