# Supplementary material for: JAK Inhibitors and B Cell Function: A Comparative Study of Their Impact on Plasma Cell Differentiation, Cytokine Production, and Naïve B Cell Activation
Source: Eur J Immunol. 2025 Mar 17;55(3):e202451437. doi: 10.1002/eji.202451437 (PMC11914861; doi:10.1002/eji.202451437)
Supplement: Supplementary file 1 — Supporting information [file EJI-55-e202451437-s001.pdf]

SUPPORTING INFORMATION

Supporting Information, Materials and Methods

Samples from healthy donors

Blood samples from healthy donors were obtained from Karolinska hospital (Details of age and gender is provided in supplementary Table 1). Peripheral blood mononuclear cells (PBMCs) were purified from buffy coats with Ficoll-Paque (Cytiva). All experiments have obeyed the declaration of Helsinki. This study was performed in accordance with the regional ethics review board (Karolinska University Hospital, Stockholm).

JAK inhibitors

Tofacitinib citrated (PZ0017) was purchased from Merck (Darmstadt, Germany), upadacitinib (29706), baricitinib (16707), and filgotinib (17669) were purchased from Cayman Chemical. All JAK inhibitors were firstly dissolved in dimethyl sulfoxide (DMSO) and later diluted in sterile water.

B cell stimulation assays

For experiments evaluating plasma cell differentiation, PBMCs (2,5x105 cells/well) were cultured with 1 μg/mL R848 (Invivogen), 10 ng/mL human recombinant IL-2 (Peprotech), and 50 ng/mL human recombinant IL-21 (Peprotech) for 3-4 days in completed RPMI 1640 medium supplemented with 10% fetal bovine serum (FBS) and penicillin-streptomycin (Sigma-Aldrich) and with different doses of JAK inhibitors (upadacitinib, baricitinib, tofacitinib, at 10, 100, 300, or 1000 nM; filgotinib at 300, 1000, 5000, 10000nM).

For experiments evaluating the activation of naïve cells, PBMCs were cultured for 2 or 4 days with completed RPMI medium and stimulated with or without 3 μg/ml anti-human-IgM (Jackson ImmunoResearch) and 50 ng/mL IL-21 together with upadacitinib at 1000nM.

For experiments evaluating cytokine expression, PBMCs were cultured in complete RPMI medium and stimulated with 1 μg/mL R848, IL-2 10 ng/mL, 50 ng/mL IL-21 for 6, 12 , 24 or 48 hours, with addition of phorbol 12-myristate 13-acetate (PMA; 50 ng/mL), ionomycin (1 μg/mL), and Brefeldin A (BFA) in the final 4 hours of culture before harvest.

Spectral flow cytometry

B cells were phenotyped by spectral flow cytometry using antibodies shown in Supporting Information Table 2-3c (SI Table 2 for general makers; SI Table 3 for intracellular cytokine panel). The B cell gating strategy is shown in SI Figure 1 and 8.

For intracellular staining, cells were cultured as described above and harvested by centrifugation. Cells were stained with Live/Dead (Zombie NIR 1:2000 Biolegend) for 15 min at 4 °C, washed with 1% FBS in PBS, and subsequently surface stained with the antibody cocktails in Supplementary table 3 and incubated at 4 °C for 30 min in the dark. Cells were fixed with BD fixation/permeabilization kit (BD Biosciences) at 4 °C for 20 min, washed two times with perm wash buffer, followed by incubation with cytokine-specific antibodies in perm/wash buffer at 4 °C for 30 min in the dark. After washing, cells were resuspended in 300 µl PBS and acquired on using a Cytek Aurora 5L instrument.

ELISA for detecting immunoglobulin G/A concentrations

High-binding half-area plates (Corning) were coated in with 2 µg/ml F(ab)2 goat anti-human IgG or anti-IgA F(ab')2 (Jackson ImmunoResearch) in PBS, followed by blocking with 1% BSA in PBS for 1h. Cell supernatants were diluted in PBS and immunoglobulin levels were detected using HRP conjugated goat F(ab')2 anti-human Ig, gamma or alpha specific (Jackson ImmunoResearch) and plates were developed using TMB substrate and stopped with 0,5 M H_2SO_4. Absorbance was measured at 450nm, with a reference filter at 650 nm. The concentrations were quantified using a standard curve of chromopure human IgG or IgA whole molecule (Jackson ImmunoResearch) with two-fold serial dilutions starting at 100 ng/ml IgG or 225 ng/ml IgA.

Western blot for detecting STAT5 and STAT3 phosphorylation

B cells were purified from PBMC using negative enrichment (B Cell Isolation Kit II Miltenyi Biotec) and 1×10^6 per condition were incubated over night with 1 µg/ml R848 with and without different JAKi before cytokine stimulation (IL-2, IL-21 or IL-2+IL-21, for 20 min). Cells were lysed in 100 µl ice-cold RIPA lysis Buffer (Thermo Fisher Scientific cat#89900) containing 1x Halt™ protease and phosphatase inhibitor cocktail (Thermo Fisher Scientific cat# 78440). Protein concentration in the lysates was determined using DC protein assay (Bio-Rad). For each lane on the Western blot, an equal amount of total protein (13 µg) was denatured and separated on SDS-PAGE with Bolt™ bis-tris plus 4-12% gels using MES running buffer

(Thermo Fisher Scientific) followed by transfer onto a PVDF membrane. Membranes were blocked with 5%. milk in PBS and incubated overnight 4°C with primary antibodies (1:1000 dilution; Cell Signaling Technology: detection of phospho-stat3 (Tyr705) (D3A7) #9145; stat3 #9132; phospho-stat5 (Tyr694) (C11C5) #9359; or stat5#94205) followed by HRP conjugated anti-rabbit IgG secondary antibody (1:2000 dilution; Cell Signaling Technology: #7074) and development with ECL substrate (Bio-Rad). Densitometric analysis was performed using ImageJ software (NIH, Bethesda, MD, USA).

Visualization of flow cytometry data

FCS files and the correction of compensation were performed on FlowJo. Unifold manifold approximation and projection (UMAP) plugin were used to analyze the flow cytometry data for visualization of phenotypic marker expression on different B cell populations after JAKi treatment. FlowSOM and clustering using cluster explorer was used to further identify different cell subsets (55). In parallel, we also used manually gating to assign cell subsets based on different markers combinations.

Statistics

Statistical analyses were performed on subsampled populations which were analyzed by one-way ANOVA with mixed effects analysis (Dunnett's multiple comparisons test or Turkey's multiple comparison test) or Two-way ANOVA with mixed effects analysis (Bonferroni test). P-values <0.05 were considered statistically significant.

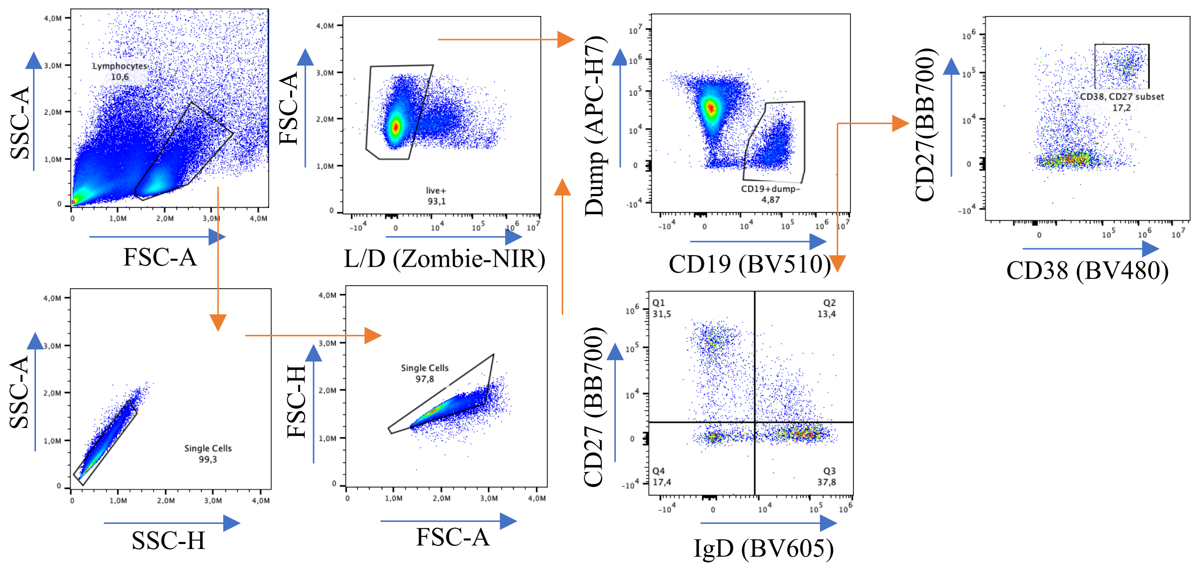

SI Fig 1. Overall gating strategy for general B-cell subsets

The figure shows an example of the gating strategy to identify different B-cell subsets by flow cytometry after in vitro culturing and stimulation. Initial gating was on FSC-A vs. SSC-A to select cells and exclude debris. This was followed by doublet exclusion using SSC-H vs. SSC-A and live/dead discrimination using L/D NIR (Live/Dead Near Infra-Red) staining to select live cells. CD19+ B cells were gated by CD19+ dump-. Plasma blasts were identified (CD38^{high} CD27^{high}) within the CD19+ population. Further characterization of non-plasmablast B cells were based on IgD and CD27 expression: naïve B cells (IgD+ CD27-); unswitch memory B cells (IgD+ CD27+), switch memory B cells (IgD- CD27+), and double negative (DN) B cells (IgD- CD27-). SSC-A; side scatter area, SSC-H; side scatter height, FSC-A; forward scatter area, FSC-H; forward scatter height, L/D; live/dead; NIR; near infra-red.

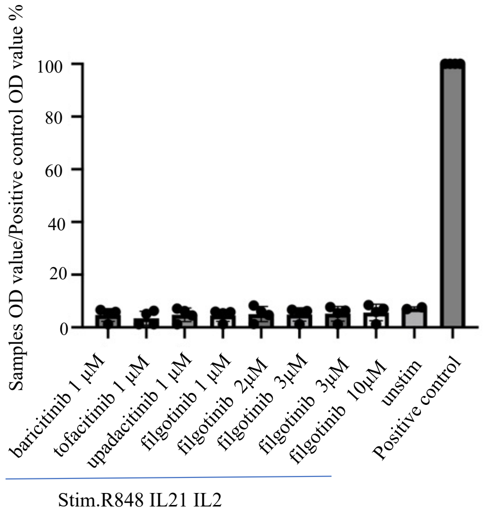

SI Fig 2. JAK inhibitors did not induce cytotoxicity under the evaluated culture conditions.

Lactate dehydrogenase (LDH) detection using a commercial assay (Promega) for cytotoxicity in PBMC cultures after R848, IL-21, IL-2 stimulation for four days with addition of JAK inhibitors at indicated concentrations. The bar graph represents the percentage of the sample's OD value towards the positive control (that generates 100% cytotoxicity). Notably, no JAKi conditions generated any significant cytotoxicity.

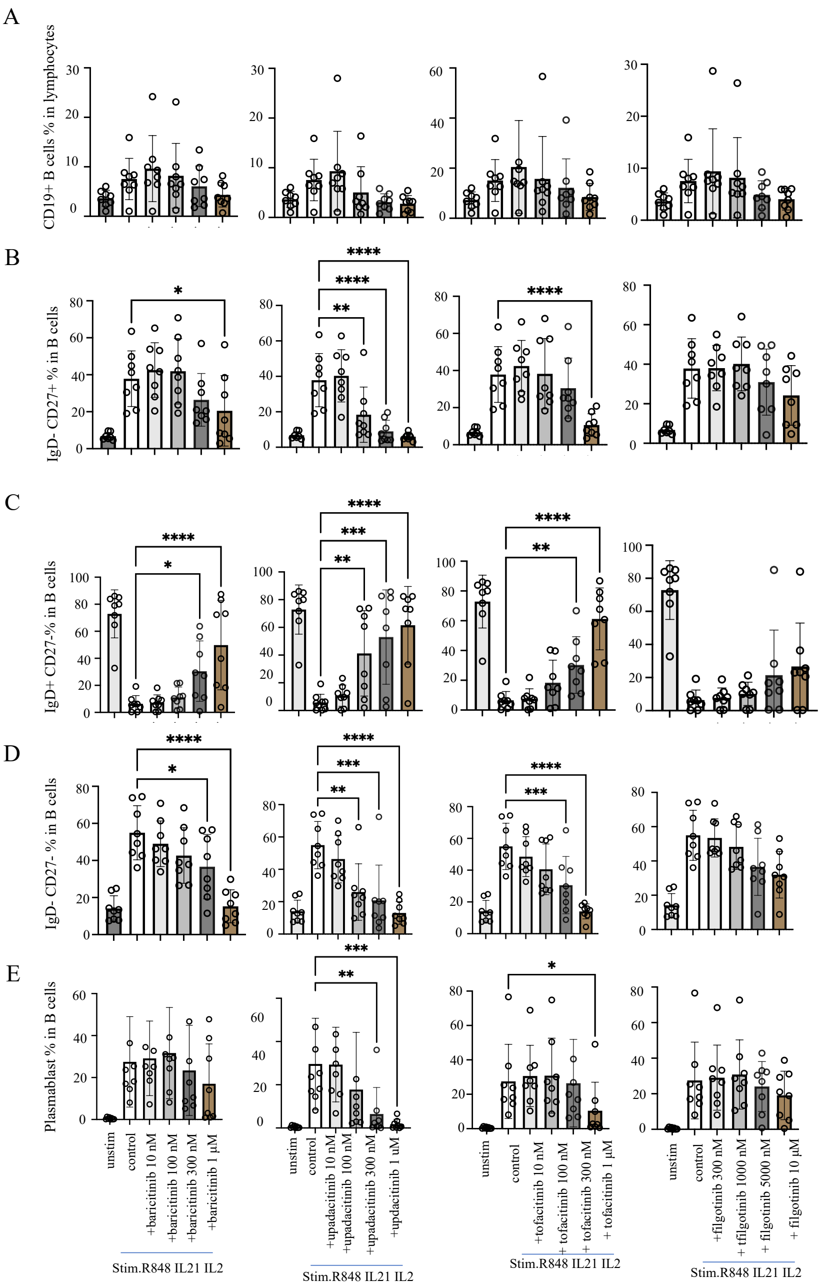

SI Fig 3. JAK inhibition of B-cell differentiation in vitro cultures.

The figure shows individual data from the flow cytometry analysis of PBMC from healthy donors (n=8), stimulated with R484 IL-21 and IL-2 in the presence of JAKi or DMSO vehicle (control). B cell phenotypes were evaluated by flow cytometry after 4 days of cultures. Figure S3 complements the average frequencies and fold changes presented in Figure 1. (**A**) Percentage of all CD19+ B cells; (**B**) Percentage of IgD-CD27+ memory B cells; (**C**) Percentage of IgD+ CD27- naïve B cells; (**D**) Percentage of IgD- CD27- double-negative B cells; (**E**) Percentage of CD27^{high} CD38^{high} plasmablast in the cultures. Different concentration of JAKi (baricitinib, tofacitinib, upadacitinib and filgotinib) were used as indicated. All data are shown as bar graphs of average values with error bars for standard deviation (SD). Group comparisons were analyzed by one-way ANOVA with Turkey method for adjusting for multiple comparisons. Statistically significance is show for the comparison between control and given the JAKi treatment group. p-values <0.05 are presented. *: p-value<0.05.**: p-value <0.01. ***: p-value < 0.001.

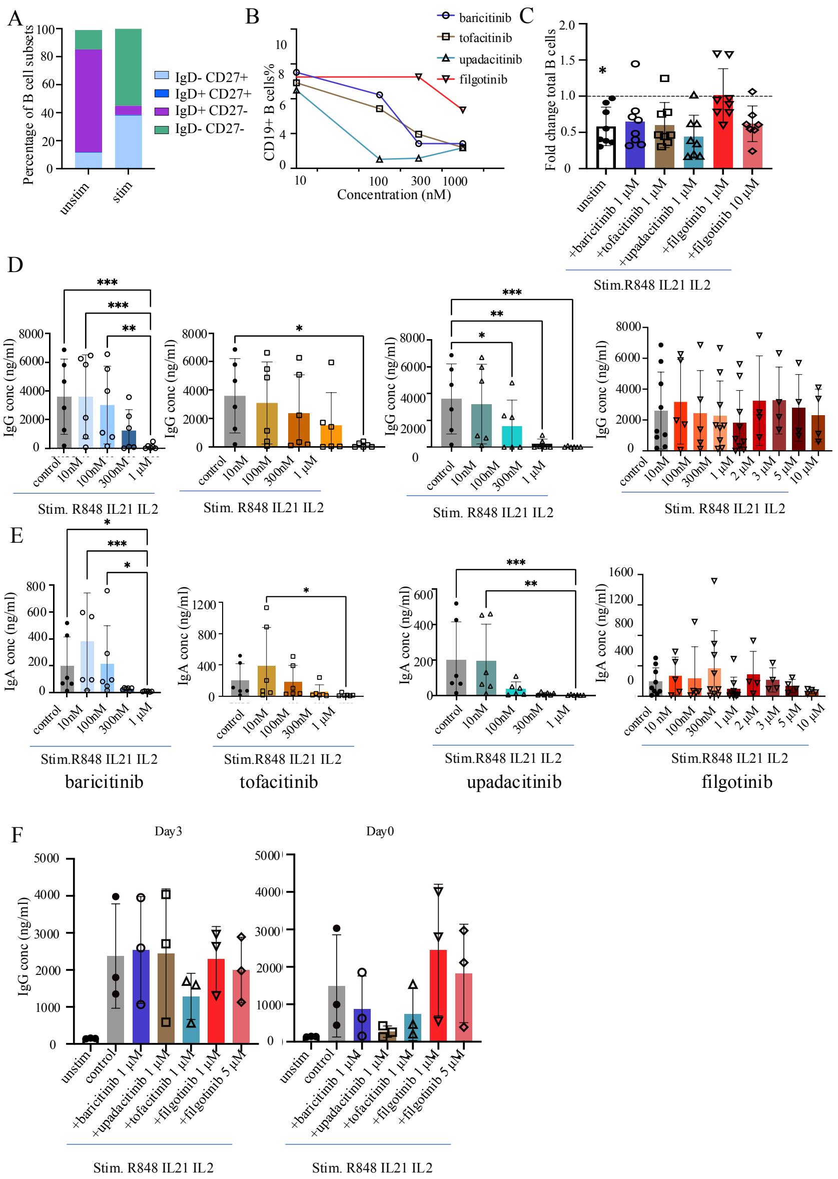

SI Fig 4. Effects of JAK inhibitors on B-cell subsets and immunoglobulin levels after 4 days of stimulation.
(**A**) Percentage distribution of different B cell subsets (IgD+ CD27+, IgD+ CD27-, IgD- CD27+, and IgD- CD27-) at day 4 following stimulation with R848, IL-2, and IL-21 (n=8). (**B**) Dose-response patterns of CD19+ B cell populations under different JAKi treatments at varying concentrations. (**C**) Fold change in CD19+ B cell percentage comparing cells stimulated in the presence of JAK inhibitors versus the control stimulated group (R848, IL-2, IL-21, and DMSO) (n=6). IgG (**D**) and IgA (**E**) levels detected by ELISA in cell culture supernatants after 4 days of PBMC culture with R848, IL-21, IL-2, with or without JAK inhibitors at the indicated concentrations. (**F**) IgG levels detected by ELISA in cell culture supernatants after 4 days of PBMC culture with R848, IL-21, IL-2, with or without JAK inhibitors added at day 3 (left) or day 0 (right) at the indicated concentrations. Bar graphs represent mean ± SD. Statistical significance was assessed by one-way ANOVA with Tukey's multiple comparison test for (C) and Dunnett's multiple comparison test for (D, E). Only p-values <0.05 are presented. *: p-value<0.05.**: p-value <0.01. ***: p-value < 0.001.

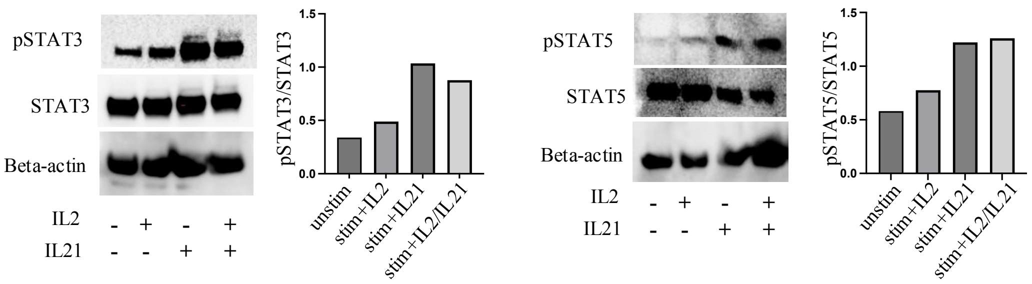

SI Fig 5. STAT3 and STAT5 phosphorylation comparing IL2 and IL21 stimulation.

Western blot analysis of pSTAT3/ STAT3/ pSTAT5/ STAT5 from purified B cells after 20min IL-2, IL-21 or IL-2+IL-21 stimulation. Purified B cells 1 × 10^6 per condition were pre-isolated from PBMCs the day before and given 1 μg/ml R848 incubation before cytokine stimulation. Cell lysate was analyzed by Western blot and changes in pSTAT3, STAT3, pSTAT5, STAT5 protein was assessed. Beta-actin was used as additional loading control in all experiments.

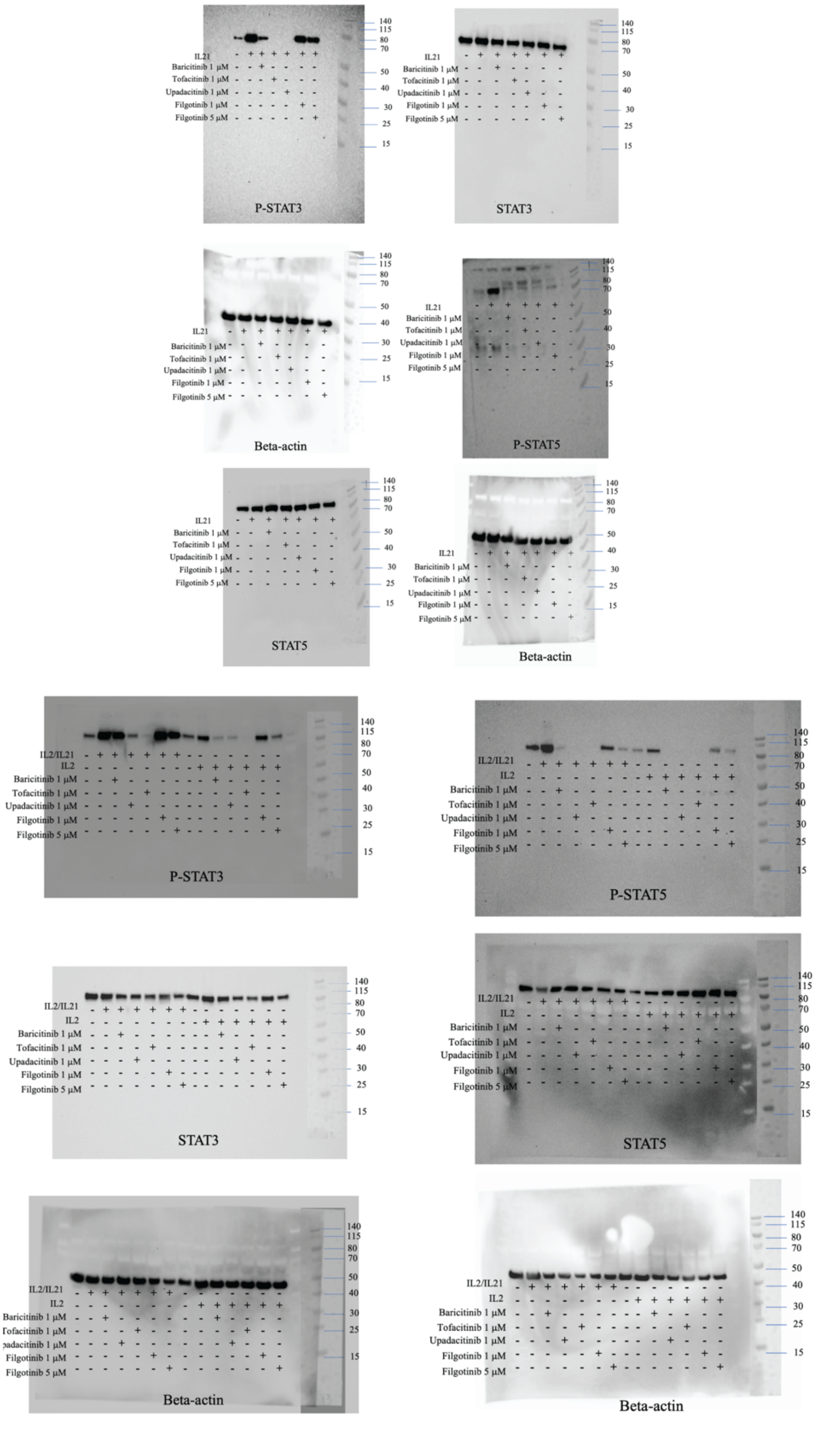

SI Fig 6. Western blot STAT3 and STAT5 phosphorylation in B cells after IL2/IL21

Uncropped Western blot membranes complementing data in Figure 2. Western blot analysis of pSTAT3/ STAT3/ pSTAT5/ STAT5 from purified B cells after 20min IL-2, IL-21 or IL-2+IL-21 stimulation. Purified B cells 1 × 10^6 per condition were pre-isolated from PBMCs the day before and given 1 μg/ml R848 incubation before cytokine stimulation. Cell lysate was analyzed by Western blot and changes in pSTAT3, STAT3, pSTAT5, STAT5 protein was assessed. Beta-actin was used as additional loading control in all experiments. STAT3/pSTAT3 and STAT5/pSTAT5 were assessed on different membranes with the same lysates. Representative data is shown.

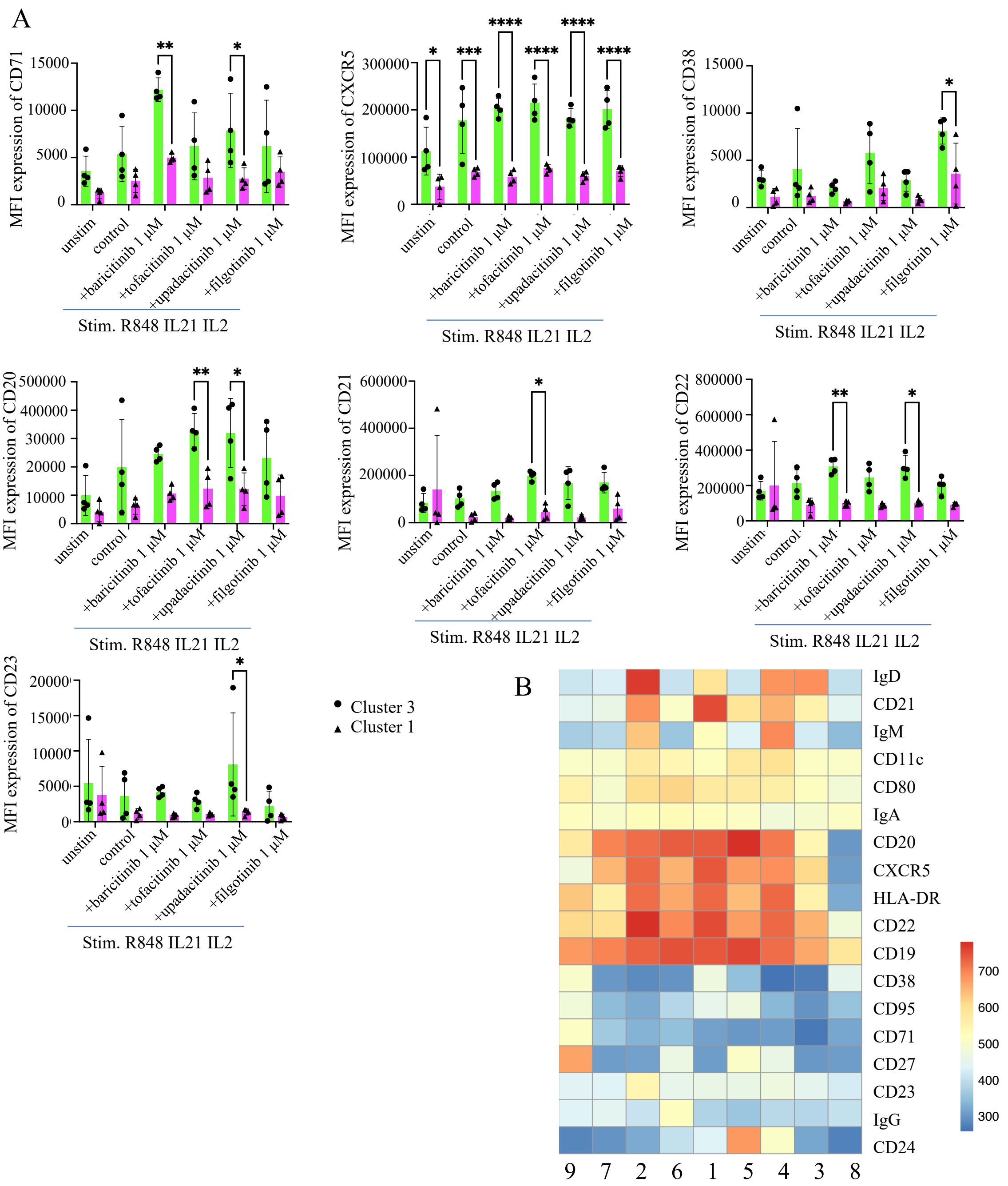

SI Fig 7. MFI expression of markers in cluster 1 and cluster 3

Comparative analysis of surface marker expression in two clusters of naïve B cells identified in FlowSOM analysis. Cluster 1 was more prevalent in unstimulated cells while cluster 3 was more prevalent in R484, IL-21 and IL-2 stimulated cells in the presence of the JAKi baricitinib, tofacitinib or upadacitinib. (**A**) MFI expression of markers (CD71, CXCR5, CD28, CD20, CD21, CD22, CD23) in cluster 1 and cluster 3 from Figure 3A. Two-way ANOVA, corrected for multiple comparisons using the Bonferroni method. Only statistically significant p-values <0.05 are presented. (**B**) The identity of each cluster in UMAP was visualized by FlowSOM heatmap showing the relative MFI expression levels of indicated markers (n=4).

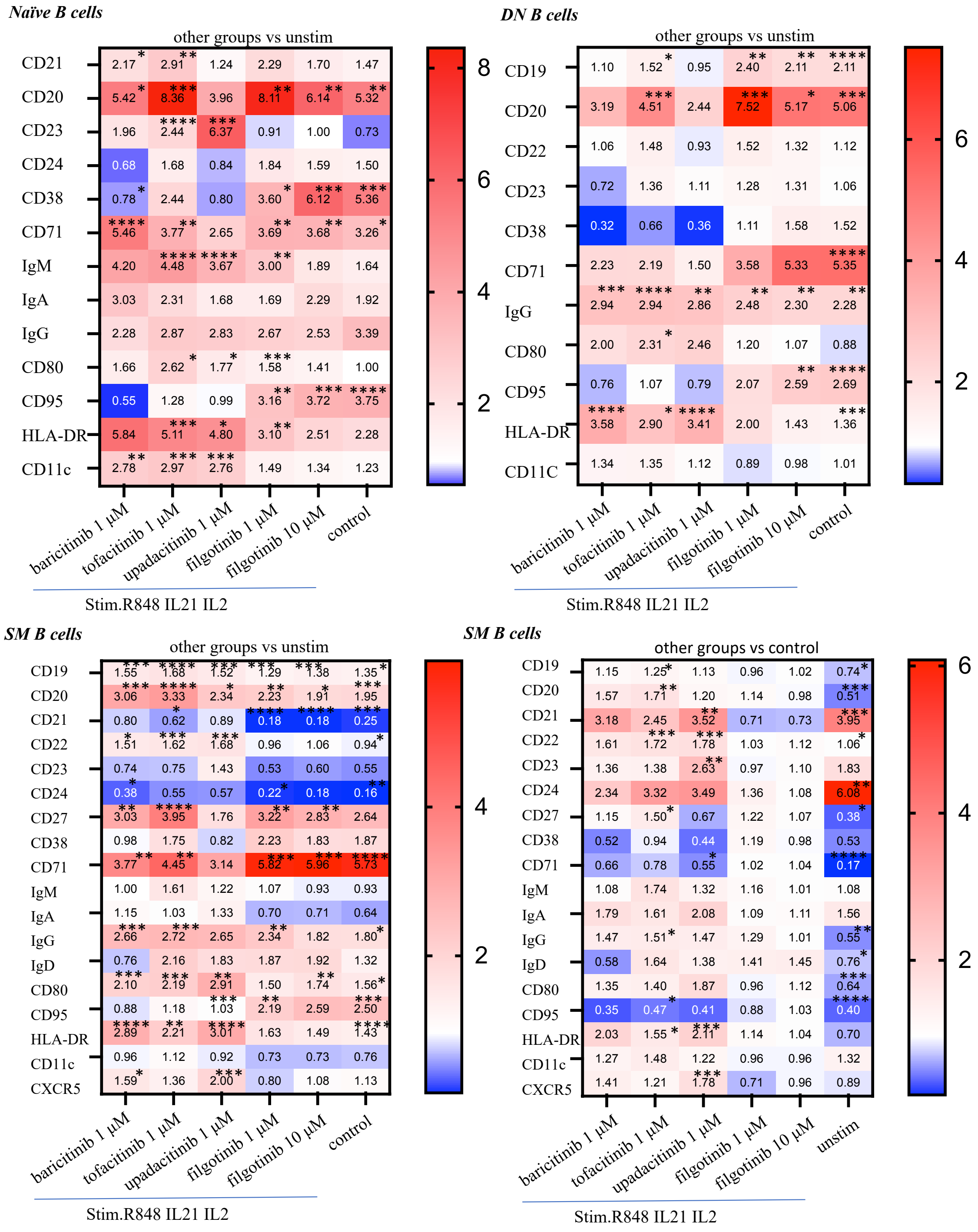

SI Fig 8. JAKi effect on surface markers of different B cell subsets.

Fold change comparison of different markers (MFI value) in CD27- IgD+ naïve B cells, CD27- IgD- double negative B cells and CD27+ IgD- switch memory B cells in in vitro PMBC cultures after 4 days of stimulation. The figure illustrates fold changes for different groups versus the unstimulated condition (naïve and DN B cells and SM B cells at left) or the IL-2 IL-21 R848 stimulated control condition (SM B cells at right)

All data were shown as heatmap with red color indicating a fold-change over 1 and blue color indicating a fold decrease (<1). Different markers MFI value (mean ± SD) in different cell subset comparisons were analyzed by one-way ANOVA with Turkey method for adjusting for multiple comparisons. Only statistically significant p-values <0.05 are presented. *: p-value <0.05.**: p-value <0.01. ***: p-value < 0.001.

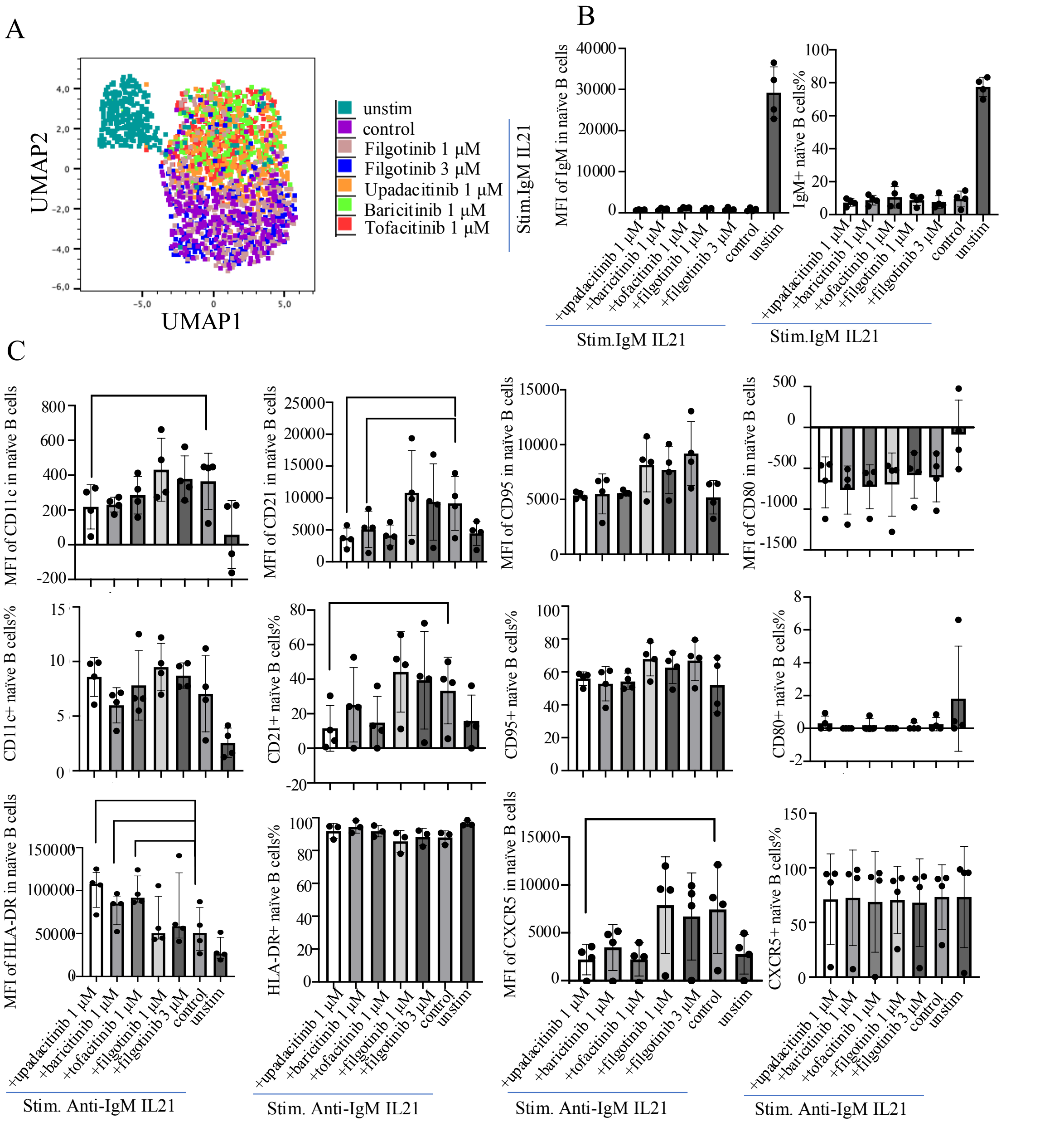

SI Fig 9. JAKi effect on naïve B cell activation.

Cultures of PBMCs from healthy donors were stimulated with anti-human IgM and IL-21 for 4 days with addition of JAKi at indicated concentrations. CD19+ B cells gated on CD19+dump-. Naïve B cells was further characterized based on IgD and CD27 expression (IgD+ CD27-). (**A**) UMAP analysis of naïve B cells in different conditions including unstimulated cells, stimulate control cells and the stimulated cells with different JAKi. Baricitinib, tofacitinib, and upadacitinib were given at 1000 nM (1 µM) and filgotinib at 1000 nM and 3000 nM (1 µM and 3 µM). (**B**) MFI values and the frequencies of IgM in naïve B cells under the conditions mentioned above. (**C**) MFI values and frequencies of CD11c, CD21, CD95, CD80, HLADR and CXCR5 in naïve B cells under the conditions mentioned above. Data in B, C data are shown as bar graphs representing average ± SD. Different markers MFI value in different cell subset were compared by one-way ANOVA analysis with Turkey's method for adjusting for multiple comparisons. Only statistically significant p-values <0.05 are presented.

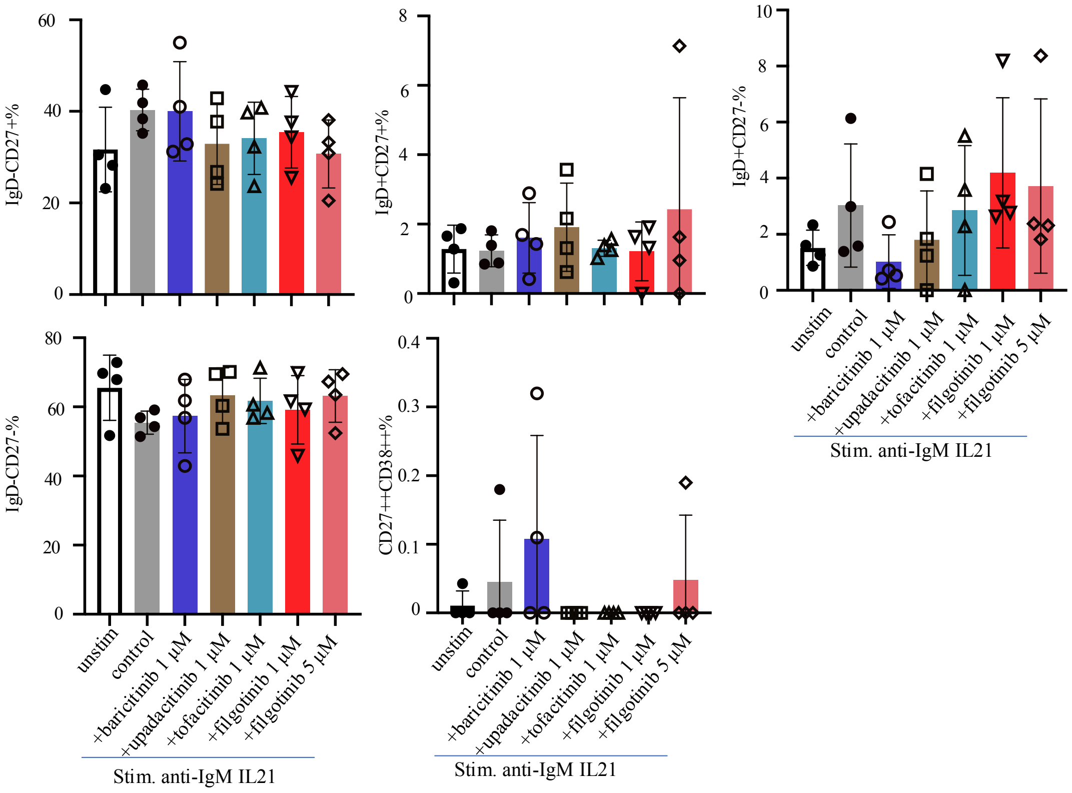

SI Fig 10. JAKi effect on B cell populations under anti-human IgM stimulation.

PBMCs from healthy donors were cultured with anti-human IgM, IL-21 with and without addition of JAKi at indicated concentration. Change in patterns of different B-cell populations among all CD19+ B cells with JAKi treatment. B-cell population shown in the graph include IgD+ CD27- naïve B cells, IgD- CD27- double negative B cells, IgD- CD27+ memory B cells, IgD+ CD27+ unswitched memory B cells and CD38^{high} CD27^{high} plasmablasts. Average of four donors are shown.

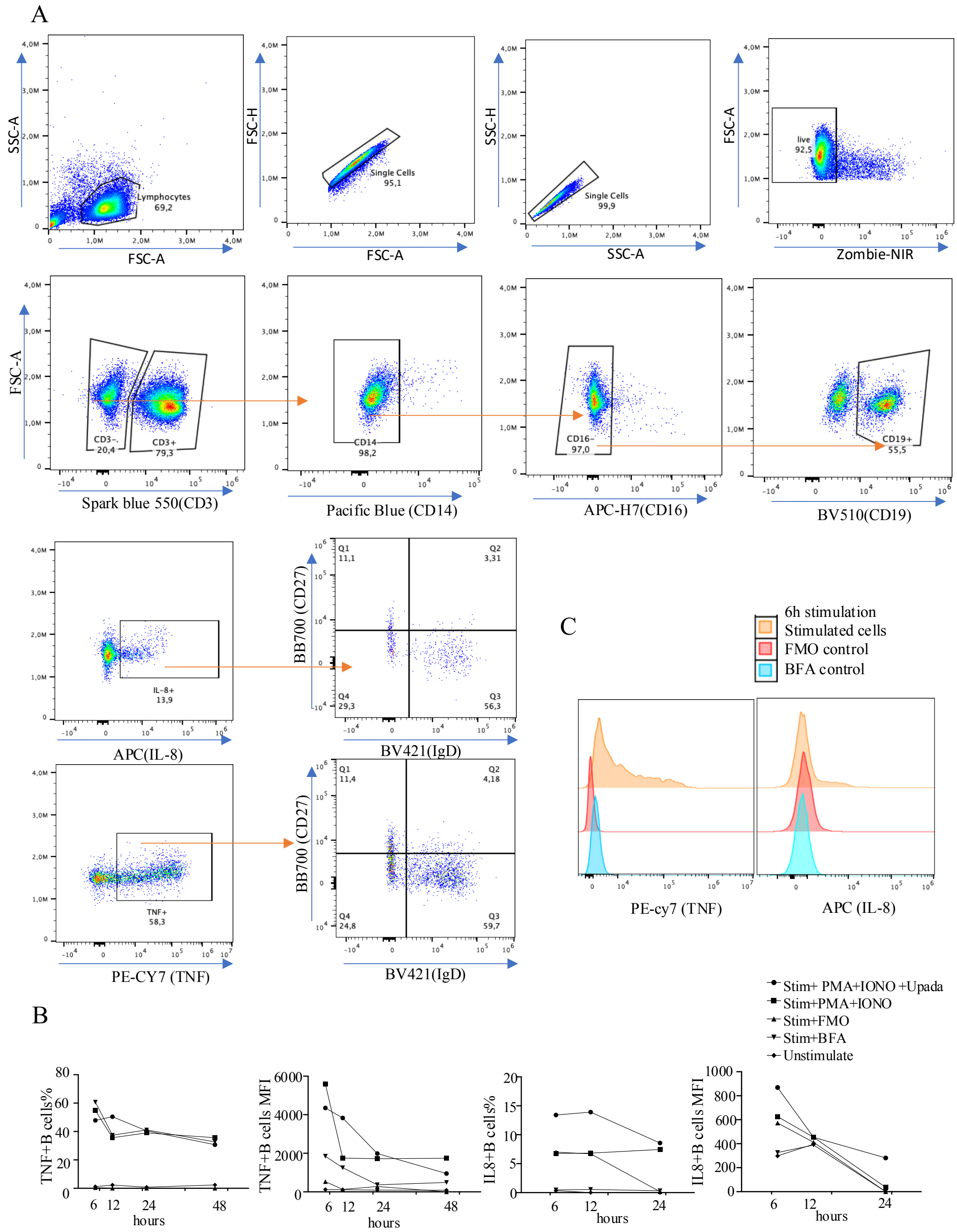

SI Fig 11. Gating strategy for intracellular flow cytometry to assess cytokine expression in B cells

The first gate is set on forward scatter (FSC) and side scatter (SSC) to exclude debris and dead cells.

A gate is set on CD19+ cells to specifically analyze B cells (After excluding the CD3+, CD14+, CD16+cells). Within the CD19+ B cell population, further gates are set to identify TNF+ and IL-8+ cells based on their fluorescence intensity. (**A**) The plot shows the gating strategy of TNF+ B cells and IL8+ B cells. (**B**) Percentage and MFI of TNF+ CD19+ B cells and IL-8+ CD19+ B cells after R848, IL-2, IL-21 stimulation at different timepoints (6h, 12h, 24h, 48h). Stim: R848, IL-2, IL-21 stimulation; PMA/IONO: PMA, ionomycin, brefeldin A; FMO, Fluorescence Minus One: Control without the TNF or IL-8 staining; Stim+BFA: After R848, IL-2, IL-21 stimulation, only BFA was given. (**C**) TNF and IL8 MFI expression in CD19+ B cells.

Supporting Information Table 1. Age and gender of the donors

Age	Gender
Plasmablast differentiation	
**Age**	**Gender**
29	Female
35	Female
61	Male
52	Female
34	Female
30	Male
25	Female
45	Male
Intracellular cytokines detection	
**Age**	**Gender**
30	Male
50	Female
38	Male
49	Female
55	Female
44	Male
57	Female
45	Female

Supporting Information Table 2. Antibodies panel used in spectral flow cytometry analysis

Antibody	Fluorophore	Clone	Company	Dilution	Catalogue No.	Instrument
Fixable viability dye	Zombie NIR		Biolegend	1/2000	423107	Cytek Aurora 4L
CD20	Pacific Blue	2H7	Biolegend	1/400	302320	Cytek Aurora 4L
HLA-DR	BV650	G46-6	BD	1/400	564231	Cytek Aurora 4L
CD22	BV711	HIB22	BD	1/400	740775	Cytek Aurora 4L
CXCR5	AF647	RF8B2	BD	1/200	558113	Cytek Aurora 4L
CD27	BB700	O323	BD	1/200	751683	Cytek Aurora 4L
CD14	APC-H7	MφP9	BD	1/200	641394	Cytek Aurora 4L
CD16	APC-H7	3G8	BD	1/200	560195	Cytek Aurora 4L
CD23	BV750	EBVCS-5	BD	1/200	747476	Cytek Aurora 4L
CD3	APC-H7	SK7	BD	1/200	560176	Cytek Aurora 4L
CD11c	PE-cy7	B-ly6	BD	1/100	742005	Cytek Aurora 4L
CD38	BV480	HIT2	BD	1/100	566137	Cytek Aurora 4L
CD21	AF700	Bu32	Biolegend	1/100	354918	Cytek Aurora 4L
CD19	BV510	SJ25C1	BD	1/100	562947	Cytek Aurora 4L
CD95	APC	DX2	Biolegend	1/100	305612	Cytek Aurora 4L
IgA*		G18-1	BD	1/50	555886	Cytek Aurora 4L
CD71	BV421	M-A712	BD	1/50	562995	Cytek Aurora 4L
IgM	BV570	MHM-88	Biolegend	1/50	314517	Cytek Aurora 4L
IgD	BV605	IA6-2	Biolegend	1/50	348232	Cytek Aurora 4L
CD24	BB515	ML5	BD	1/50	564521	Cytek Aurora 4L
IgG**		G18-145	BD	1/50	555784	Cytek Aurora 4L

*IgA was conjugated using NovaFluor Blue 610-70S conjugation kit (ThermoFisher, #K06T04L011).

**IgG was conjugated using AF532 conjugation (ThermoFisher, #A20182).

Supporting Information Table 3. Antibodies panel of intracellular staining of cytokine expression

**Antibody**	**Fluorophore**	**Clone**	**Company**	**Dilution**	**Catalogue No.**	**Instrument**
CD3	sparkblue 550	SK7	Biolegend	1/100	344852	Cytek Aurora 5L
CD19	BV510	SJ25C1	BD	1/100	562947	Cytek Aurora 5L
CD16	APC-H7	3G8	BD	1/200	560715	Cytek Aurora 5L
CD14	Pacific blue	M5E2	Biolegend	1/200	301828	Cytek Aurora 5L
CD27	bb700	O323	BD	1/100	751683	Cytek Aurora 5L
IgD	BV421	IA6-2	BD	1/100	562518	Cytek Aurora 5L
HLA-DR	Percp cy5,5	G46-6	BD	1/200	560652	Cytek Aurora 5L
CD24	BB515	ML5	BD	1/50	564521	Cytek Aurora 5L
CD38	AF594	HIT2	Biolegend	1/50	562288	Cytek Aurora 5L
Live/dead	Zombie nir		Biolegend	1/2000		Cytek Aurora 5L
**Intracellular**						
**Antibody**	**Fluorophore**	**Clone**	**Company**	**Dilution**	**Catalogue No.**	
IL8	APC	8CH	Invitrogen	1/50	2345019	Cytek Aurora 5L
TNF-a	PE-cy7	MAb11	Biolegend	1/50	502930	Cytek Aurora 5L

Supporting Information Table 4. Gating strategies

B cells subsets	***Gating strategy***
IgD-CD27- B cells (DN B cells)	Lymphocytes/Single Cells/Single Cells/live+/CD19+CD3-CD14-CD16-/IgD-CD27-
IgD+CD27- B cells (naïve B cells)	Lymphocytes/Single Cells/Single Cells/live+/CD19+CD3-CD14-CD16-/IgD+CD27-
IgD-CD27+ B cells (memory B cells)	Lymphocytes/Single Cells/Single Cells/live+/CD19+CD3-CD14-CD16-/IgD-CD27+
IgD+CD27+B cells	Lymphocytes/Single Cells/Single Cells/live+/CD19+CD3-CD14-CD16-/IgD+CD27+
Plasmablast	Lymphocytes/Single Cells/Single Cells/live+/CD19+CD3-CD14-CD16-/CD38++CD27++
